# Plant Invasion Research in Russia: Basic Projects and Scientific Fields

**DOI:** 10.3390/plants10071477

**Published:** 2021-07-19

**Authors:** Yulia K. Vinogradova, Valeriy K. Tokhtar, Alexander A. Notov, Sergey R. Mayorov, Elena S. Danilova

**Affiliations:** 1 Laboratory of the Native Flora, N.V. Tsitsin Main Botanical Garden RAS, 4 Botanicheskaya St., 127276 Moscow, Russia; gbsad@mail.ru; 2 Botanical Garden, Belgorod State National Research University, 85 Pobedy St., 308015 Belgorod, Russia; 3 Faculty of Biology, Tver State University, 33 Zhelyabov St., 170100 Tver, Russia; anotov@mail.ru; 4 Faculty of Biology, M.V. Lomonosov Moscow State University, 1 Leninskie Gory, Building 12, 119234 Moscow, Russia; saxifraga@mail.ru; 5 Department of English Philology and Cross-Cultural Communication, Belgorod State National Research University, 85 Pobedy St., 308015 Belgorod, Russia; elena_danilova@bsu.edu.ru

**Keywords:** alien plant species, invasion, invasion biology, Russia, habitat, flora, cultivation

## Abstract

This paper represents a review of comprehensive research into invasion biology done by Russian scholars for four decades. Invasion biology is a relatively new field of research focused on the study of alien plants, their behavior in new habitats and risks they pose to indigenous species. It is shown that over 40 years, single finds of alien plant species have transformed into a check-list of invasive species in Russia. The most significant invasion pathways were identified, and the rate of microevolutional changes in plant species in their secondary distribution range was determined. Since the most active alien species disperse without regard to national borders, international cooperation is crucial in joint studies of invasive species.

## 1. Introduction

Invasion biology is a rather new field of research which addresses the ability of alien species to expand the secondary distribution range and invade natural ecosystems, as well as the factors and consequences of alien species introduction, their impact on ecosystems, potential advantages and risks they pose. The founder of the “bioinvasion” science was Charles Elton. Discussions on this phenomenon may also be found in works by other scholars. Ch. Darwin wrote: “No one can tell until he tries, whether…any exotic plant seed freely under culture…Lastly, when organic beings are placed during several generations under conditions not natural to them, they are extremely liable to vary” [1] (Chapter VIII, p. 234). However, it was C. Elton who generalized the disparate data and used the term “invasion” in the title of his book [2].

Over the past 200 years, flora and fauna have changed significantly: in some ecosystems, alien (non-native) species which have naturalized in their new homelands outnumber native ones. Biological invasions threaten the biological diversity of the planet [3]. It is important to describe how the science of biological invasion developed in Russia. In this paper, we will focus on plant invasion.

At the first stage of invasion biology development as a scientific discipline (1927–1988), research objects (alien species and invasive species) were identified and a targeted search for new alien plant species began. At the second stage (1989–2002), numerous lists of “adventive floras” were compiled and microevolution patterns displayed by alien species in the secondary distribution range were identified. At the third stage (2003–2014), the study focus shifted from all alien species to invasive ones. The current fourth stage (since 2015) is characterized by the completion of a general invasive species inventory. This allows for a detailed study of priority species (transformers) biology and monitoring of invasive populations to predict their further expansion.

The science of invasion biology is now developing rapidly in Russia. It encompasses various aspects of studying invasive species and comprehensive issues of biology and ecology, including biogeography, systematics, introduction, and microevolution research. A number of new independent lines of research have emerged which are focused on the study of alien species and biological invasions. Many of them have already yielded fruitful results.

The aim of this review is to summarize preliminary results of invasion biology development in Russia. Special attention will be paid to the lines of research which have already emerged, as well as to the findings and prospects. We will begin the review with the description of general trends and focus on their specifics in Russia. Further in our paper, we will discuss particular cases which reflect the research interests of Russian scholars.

## 2. Materials and Methods

The academic sources used for this review included research papers on various aspects of the study of alien and invasive species, abstracts of meetings, PhD theses, and proceedings of conferences held from 1989–2020. As invasion biology developed in Russia, the scope of research expanded significantly. Studying new aspects of research into alien species and invasive flora fraction contributed to new approaches to their analysis. Authors of this paper have largely participated in developing such approaches. Methodological specifics of the latter are detailed below in the descriptions of separate lines of research.

## 3. Results and Discussion

Russian scientists are studying various aspects of alien plant species biology. According to the Aichi Biodiversity Target 9, botanists in Russia are working towards identification and prioritization of invasive alien species and pathways, controlling or eradicating priority species, and managing pathways to prevent their introduction and establishment. Since 2008, the Russian Journal of Biological Invasions and its Russian-language version (www.sevin.ru/invasjour/ accessed on 24 June 2021) have been published in Russia. They address urgent issues of invasion biology and assess risks for native ecosystems posed by invasions. By 2020, 45 issues had been published. More than a hundred articles were devoted to invasion of plant organisms.

The “Invasion of Alien Species in Holartic” symposium, the only international event dedicated to alien species and held in Russia on a regular basis, played a crucial role in the development of research on biological invasions. The first symposium—Borok-1—was held in 2001, and the last one—Borok-5—in 2017. These scientific events focused on analyzing natural and anthropogenic factors of alien species settlement. Among other issues discussed were characteristics of ecosystems and the influence of invasive species on the flora and fauna [3].

### 3.1. An Inventory of Alien Plant Species

The first Russian research paper on the alien fraction of flora described as the “adventive flora” was written by M. Nazarov. The author showed that some introduced plants established very successfully during the decline in agriculture and population migration [4]. Herbarium collections of non-native species in the first half of the 20th century were still scattered, but in the 1970s, this group of plants fell within the field of interest of Yu.D. Gusev [5,6,7,8,9,10,11], V.N. Tikhomirov [12], and A.K. Skvortsov [13,14]. More papers were published after 1983, including works by V.V. Makarov, M.S. Ignatov, V.D. Bochkin, and A.V. Chichev on findings of alien plant species in the Moscow region [15,16,17,18,19], and Yu.K. Vinogradova (Maytulina) on the alien species in Vologda [20]. It was necessary to consolidate fragmentary information. In order to coordinate the research data, the meeting “Problems of Studying the Adventive Flora of the USSR” was held in Moscow. Sixty-seven scientists from Ryazan, Tula, Voronezh, Moscow, Ivanovo, Kursk, Magadan, and other regions made reports on alien plants [21]. It was proposed to use alien plants as a model for studying microevolutionary processes. The “climax” of compiling floristic lists was the work “Abstract of the alien plant flora of Moscow Region” [22]. It provided an example of generalization of data on the flora in various regions. After a long break, in 2003, the conference “Problems of Studying Adventive and Synanthropic Flora in the CIS Regions” was held in Tula.

Currently, studies are aimed at: (a) taking an inventory of alien species, especially quarantine plants, in understudied regions [23,24,25,26]; (b) identifying special aspects of alien species global migration along ecological or geographic gradients [27,28,29,30]; and (c) assessing the degree of plant invasion in protected areas [31,32,33]. The study of the global invasion of alien species is impossible without analyzing their distribution in various regions of Russia [34]. Special attention is paid to the analysis of alien flora fraction in areas with advanced industry and agriculture, such as Moscow, Belgorod, Tver, Ivanovo, Tula, Bashkiria, Udmurtia, etc. As a result, special reviews have been made for different regions [35,36,37,38,39,40,41,42,43,44,45,46,47,48] (Table 1). The study of alien species has been intensified in Siberia and in the Far East, where biologists are now developing regional “Black Books”.

Many authors stress that processes of alien species naturalization into various regions of Russia are intensifying [29,42,68,86,92]. In particular, 32 new alien species (12 of which are new to the flora of the Central Chernozem region) were found in Belgorod region from 2010 to 2021 for the first time: *Amaranthus graecizans* L. s. str., *Cenchrus longispinus* (Hack.) Femald, *Euphorbia davidii* Subils, *E. marginata* Pursh, *Grindelia squarrosa* (Pursh) Dunal, *Oenothera oakesiana* (A. Gray) Robbins. ex S. Watson, *Panicum dichotomiflorum* Michx., *Potentilla bifurca* L. [50], etc.

Macroecological studies are becoming an important area of research. A joint project implemented by the Institute of Botany of the Czech Academy of Sciences (Prague), the Main Botanical Garden of the Russian Academy of Sciences (Moscow), and the Botanical Garden of Belgorod State University is among the most large-scale ones of recent time. This project includes a joint study of alien species global migration along railroads within the Trans-Siberian Railway [83,159,160].

### 3.2. An Inventory of Invasive Plant Species

The first check-list of invasive species (for Northwest Russia) was presented by D. Geltman; he also emphasized the need for a more detailed study of this group [69].

**Figure 1:**
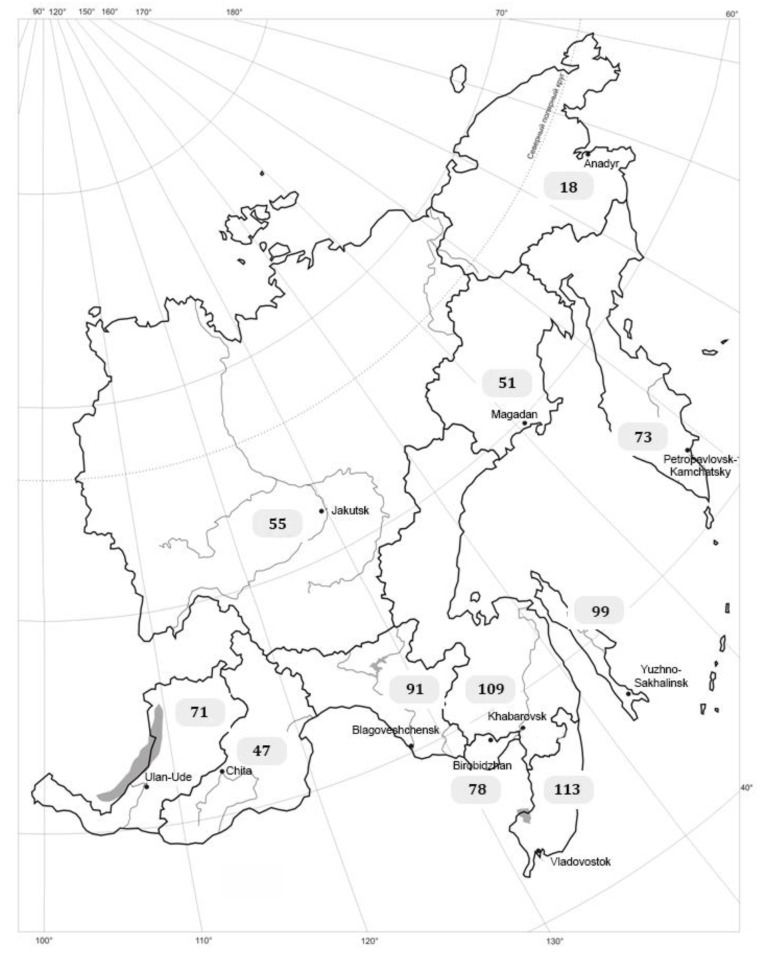
The number of invasive plant species in administrative subjects of The Far Eastern Federal District (taking into consideration currently available data (see Table 1) and original materials).

As of today, methodological aspects of creating “Black Books” and “blacklists” of particular regions have been developed taking into consideration the danger posed by invasive species and their distribution specifics according to four invasiveness statuses [161]: (1) transformers which actively invade natural and seminatural communities; change ecosystems; disrupt succession processes; act as edificators and dominants, forming single-species dense stands; and force out and (or) obstruct the reproduction of native species; (2) alien species actively spreading and becoming naturalized within disturbed, seminatural, and natural habitats; (3) alien species currently spreading and undergoing naturalization in disturbed habitats; in the course of further naturalization, some of them will apparently be able to establish themselves in seminatural and natural communities; and (4) potentially invasive species capable of propagating within places of introduction or ones which have manifested themselves as invasive species in adjacent regions. *The Black Book of Flora of Middle Russia* was published in 2010 [52]. Data on 52 invasive plant species—the most noxious ones which are widespread on the territory of Middle Russia—are presented in the book. It provides information on distribution patterns within the secondary distribution ranges for each species, as well as an estimate of economic damage and recommendations for possible usage of invasive species. Original methods to control invasive species, reduce the abundance, and decrease the distribution ranges are proposed. The “Blacklist” of 100 alien plant species has been compiled, requiring urgent studies and monitoring in order to prevent invasion. Inventory of invasive species, together with mapping their distribution ranges (maps are based primarily on herbarium data) provide an important source of information for specialists in nature protection. Currently, the “Black Books” of the following regions have been published based on the same methodology: Tver region [64], the Udmurt Republic [82], Siberia [93], Nizhny Novgorod [84,85], and Kaluga region [58]. The *Black Book of Flora in Bashkiria* and the *Black Book of Flora in the Far Eastern Federal District* are in preparation for publication. The use of unified methodology has enabled scholars to compile lists of invasive species (blacklists) of the Upper Volga region [57], the Sura River basin [28], Middle Urals [90], Yaroslavl region [67], Khabarovsk Territory [131,133], Volgograd region [80], Bryansk region [53], Voronezh region [56], the Middle Volga region [87], Kabardino-Balkarian Republic [74], and the Far Eastern Federal District [105] (Figure 1).

The list of invasive species occurring in the Far Eastern Federal District (FEFD) includes 117 taxa. Comparing the spectrum of life forms of the invasive flora fraction in the European part of Russia and the FEFD, we have found that both annual and perennial herbaceous species in the two regions display equal proportions: 38% in the European part of Russia, and 46% and 47%, respectively, in the FEFD. The difference between the regions consists in a higher proportion of woody species in the invasive fraction of the flora in the European part (24 vs. 6). We explain this by a more active intentional introduction carried out in the European part of Russia—all of the woody species have escaped from cultivation.

At the current stage, the inventory of available data on alien and invasive species of Russia has been completed. In 2015, the Blacklist of invasive plants of Russia was published by 50 scientists from 42 administrative regions. Due to the vast territory of the country, the compilation of a single list is deemed inappropriate. It includes three sections: the European part of Russia, Siberia, and the Far East. Twenty-four alien plant species are present in all Russian regions [162]. Authors have identified alien plant distribution patterns in various regions of the country, depending on the anthropogenic load on ecosystems [163].

A database of animal and plant alien species of Russia was created and patented, and is now available on the Internet [164]. The book *The Most Dangerous Invasive Species of Russia (TOP-100)* was published in 2018; it contains information on 100 plants and animals which are most dangerous for ecosystems. The authors present original maps of natural and invasive ranges, invasive corridors and pathways, biology features, effects on native species, ecosystems and humans, and abundance control methods for each species [165].

The data obtained make it possible to conduct monitoring studies of biological invasions and alien species. However, the high dynamics of the invasive component determines the need to continue the inventory work, especially in poorly studied regions.

### 3.3. Analysis of Long-Term Dynamics of Alien Flora and Its Invasive Component

In our previous studies, we developed methodological aspects of alien flora formation [46,63,65]. A quantitative estimate for the rate of alien flora invasion in Tula region was provided based on changes in the species’ naturalization degree for the last 200 years [65]. Wavelet analysis enabled an assessment of the dynamics of the alien flora in Tula region which revealed the following facts: (1) the average rate of flora enrichment with alien species has been constant for these 200 years, amounting to 15 species per decade; and (2) the average rate of naturalization has been relatively low and constant, amounting to 5 species per decade. Fluctuations in the composition and naturalization degree of alien flora species in the Tula region have not proved to directly depend on the changes in the territory’s economic development during the last two centuries [65].

The dynamics of alien flora in the Tver region for over 200 years have also been analyzed, with general trends in alien flora shifts revealed [46]. The naturalization of various alien plant groups was assessed. A detailed study of the Tver city flora for a 200-year period [63] enabled the authors to reveal the specific structure of native, alien, and invasive components at different stages of the city’s development. Furthermore, the botanical and geographical specificity of the native and alien components was determined in historical and contemporary perspectives. For 200 years, 1143 species of vascular plants have been recorded (675 native and 468 alien ones). More than 30% of the specific structure of native flora have been lost. The alien component of contemporary city flora (represented with 427 species) is comparable with the native one (479 species). The level of alien and invasive species variety has increased almost four times, and the number of protected plants has decreased more than four-fold.

During the study of the invasive flora fraction in the southwestern Central Russian Upland over a 170-year period (from 1850 to 2019), more than 200 natural habitats of the region were analyzed, and 78 invasive species were identified. The invasive component formation in the area comprised three stages: (1) intentional or unintentional introduction of alien plants (1850–1929); (2) naturalization of alien plants (1930–1989); and (3) intensification of plant introduction and invasion (1990–2019). Changes in the structure of the invasive flora component during the 170-year period are primarily associated with human economic activities and the intensity of anthropogenic impact on natural ecosystems [24].

### 3.4. Development of Methods for Alien Species Study

Currently, a significant number of methods have been developed in Russia to assess processes of increasing the amount and importance of alien plants in the flora, as well as the invasive success of some alien species. Some of them are based on traditional empirical approaches, while others rely on statistical analysis. Visualization of results has recently become important for developing predictive models of invasions [166]. Canonical correlation correspondence methods are used in order to explain the distribution patterns of closely related species. They make it possible to identify factors limiting the distribution of such species. This approach enables researchers, among others, to visualize the dependence of *Oenothera* species distribution and determine the spatial location of centroids of their ecological niches in Eastern Europe in relation to the limiting factors [167].

Much more complex issues include the identification of distribution patterns displayed by unrelated species groups which migrate conjugately to different types of natural (in the case of naturalization) and anthropogenically transformed ecotopes. In this case, promising data visualization methods include ones based on the estimation of correlation matrices with calculated similarity coefficients. These are: factor analysis, correspondence analysis, discriminant analysis, and other methods of multivariate statistics [33]. The use of multivariate statistical methods makes it possible to establish patterns of florocomplex structure formation with alien species participation in response to an increased anthropogenic impact.

In addition to multivariate statistical methods, GIS-technologies, the use of satellite monitoring data and hyperspectral remote sensing bear great prospects in understanding the migration patterns of alien plant species [33,166,168,169,170].

### 3.5. An Inventory of Polemochores

Since 2012, Russian scholars have also focused on polemochores which expanded their range in the course of military operations, for instance, during The Great Patriotic War of 1941–1945 [171,172,173,174,175]. As of today, more than 45 polemochorous species have been identified in Central Russia [176]. The specificity of their distribution in Central Russia may be explained with the influence of historical, military, and natural factors. The initial level of polemochore diversity was determined by the volume and composition of imported diasporas, which depended on the occupation duration and military operations specifics. The largest number of polemochores was found in the immediate vicinity of transit hubs connecting railroads with unpaved local roads, as well as near the locations of army depots. Horse fodder was a major source of polemochore seeds [176]. In addition, grass mixtures were apparently used in some places to camouflage positions. Generally, the type of plant communities, landscape transformation level, economic use of the area, and the peculiarities of successional vegetation dynamics are of great importance for the preservation of polemochores [177]. Polemochores are stable in meadows, clearings, and edges in light forests which are not subject to intensive economic activity and are little transformed during successional shifts. We have considered biological and ecological characteristics of polemochores in Tver region, along with succession dynamics of phytocenoses with polemochores. It enables us to make a preliminary assessment of their invasive potential [177,178]. According to our findings, the vast majority of polemochores do not tend to spread further, but some species may settle outside of their initial habitats. The results indicate that the Great Patriotic War of 1941–1945 was an important factor in the modern genesis of the flora in Central Russia [176].

### 3.6. Identification of Naturalized Species in Botanical Gardens

Guided by the resolution of the European Botanic Gardens Congress (Helsinki, 2009), the “Code of Conduct for Botanic Gardens on Invasive Alien Species” was developed [179,180]. It was adopted by the participants of the conference on biodiversity conservation (Yaroslavl, 2011) and approved at the First Organizing Congress of the Council of Botanical Gardens of the CIS countries under the International Association of the Academy of Sciences (Moscow, 2013). An analysis of the invasive potential of cultivated, weed, and alien plants found on the territory of botanical gardens was carried out at the botanical gardens of Tver [181], Voronezh [54,182], Rostov [183], and Kaliningrad [71].

Flora in the Main Botanical Garden of Russian Academy of Sciences (MBG, Moscow) were analyzed in more detail. It comprises 941 species from 427 genera, belonging to 107 families of vascular plants. This is the first time that scholars have considered the dynamics of changes in the local flora for such a long period (since 1949). The study has revealed that for 70 years, the local flora has increased 1.8 times. It has been replenished with 62 natural flora taxa, 284 species which escaped from cultivation and 36 alien weeds. *Adenocaulon adhaerescens* and, presumably, *Geum macrophyllum* Willd. have reliably “escaped” from the territory of MBG RAS. The alien component of the flora is characterized in terms of taxonomy, life-form, the type of distribution range, and invasive status. The study conducted at MBG makes it possible to identify the differentiating families of native and alien flora fractions.

It also allows us to describe botanical gardens as a trigger mechanism for introduced plant species microevolution, as well as to present data disproving the role of introduction institutions as a major pathway of biological invasions. The latter is a misconception wide-spread in academic papers [184,185].

One of The Code objectives is to alarm people on the dangers posed by alien species. It may be done by installing special information booths, creating mini-exhibitions, and promoting research results through booklets and brochures.

The Commission on Invasive Species established by the Council of Botanical Gardens of Russia, Belarus, and Kazakhstan, is working out principles to create exhibitions of invasive alien plant species. This is a new task for botanical gardens. The underlying idea of such exhibitions is bilateral and aims at:

(a) presenting noxious species in order to develop effective means of control. A provisional name for this exhibition could be “Our cultivation in the garden is strongly prohibited!” For a better effect, such an exhibition should be located directly in front of the entrance to the botanical garden. *Oenothera biennis* L. and *Conyza canadensis* (L.) Cronq. may be planted in the background; *Galinsoga parviflora* Cav., *G. quadriradiata* Ruiz & Pav., *Matricaria discoidea* DC., etc., may be planted in the front zone;
(b) presenting species which are actively turning wild within the garden territory, being potentially invasive. In fact, the species mentioned in the previous paragraph (e.g., *Conyza* and *Galinsoga*) escaped from botanical gardens a few centuries ago. A provisional name for such an exhibition could be “Do not let us escape!” *Helianthus tuberosus* L., *Solidago canadensis* L., *Echinocystis lobata* (Michx.) Torr. & A. Gray, and *Aster novi-belgii* may be planted in the background; *Oxalis stricta* L. and *Bellis perennis* L. may be planted in the front zone.

Species introduced at such exhibitions should be planted in portable containers, while juvenile plants may be simply put in containers. It is strongly prohibited to cultivate plants from seeds (especially from alien sources), as each subsequent generation adapts to a new environment much better. Containers may be placed near invasive trees and shrubs (for instance, *Acer negundo* L. or *Robinia pseudoacacia* L.) quite often cultivated in introduction centers. The main rule for composing such exhibitions is to use only the plants growing nearby, without creating new centers of invasive species distribution.

One of the key advantages of this approach is minimizing cultivation efforts: invasive species are most adapted to the changing environment. Utmost attention should be paid to colorful labels describing the dangerous invasive features of the species.

It is necessary to prohibit plants from fructification. Inflorescences and flowers should be cut down immediately upon the end of flowering. The label should contain a picture of the blossoming plant. At the end of the vegetation season, all plants have to be eliminated, instead of being wasted in compost heaps. As the first step, a prototype “micro-exhibition” of invasive species has been organized in RAS Main Botanical Garden. The exhibition comprises strictly invasive species (*Solidago gigantea* Aiton, *Bidens frondosa* L., and *Impatiens parviflora* DC.), and those actively turning wild (*Geranium phaeum* L., *Veronica filiformis* J.E. Smith, *Adenocaulon adhaerescens* Maxim., and *Geum macrophyllum*). The second group of species is potentially invasive.

### 3.7. Microevolution of Alien Species in the Secondary Distribution Range

In 1979, Yu. Maitulina (Vinogradova), guided by A. Skvortsov, began to study biological characteristics of invasive species. Since the term “invasion” was not used, the group was described as “plants belonging to different life forms, with different biology, brought to Russia at different periods and in different pathways. They have one thing in common: they undergo active naturalization and expansion of their secondary distribution range.” In her PhD thesis, the intraspecific variability of *Acer negundo, Echinocystis lobata,* and *Conyza canadensis* was studied using the method of creating experimental introduction populations from seeds of different geographical origin [186]. The seeds had been collected from the extreme northern (Arkhangelsk, Vologda) to the extreme southern (Ashgabat, Astrakhan) points of the secondary distribution range of the species. The amplitude of the clinal variability of plant bio-morphological characteristics was revealed by the gradient of soil and climatic conditions variation.

The results of studying microevolution in 20 noxious alien plant species were later summarized [187]. It was shown that microevolutionary processes in self-pollinators and cross-pollinating species follow different patterns. In the first case, a genotype develops in the initial invasive population adapted to various climatic conditions, but a morphologically homogeneous one. In the second case, classical natural selection takes place. It contributes to the intraspecific variability of plant bio-morphological characteristics.

Microevolutionary processes have also been noted in alien species with vegetative propagation. The absence of sexual reproduction does not hinder the rapid expansion of *Veronica filiformis* (Plantaginaceae). Mowing and raking of lawns cause fragmentation of *V. filiformis* shoots and increase the rate of its clonal dispersal. During the vegetation season, the diameter of a clone can increase 25 times, and the number of internodes—by a factor of 1–2 thousand! From the point of its initial introduction (Moscow), *V. filiformis* settles at the rate of 4 km/year to the north and 10 km/year to the south. The ability of the species to regenerate is more influenced by the lighting intensity than by the age of the rooting shoots. In the last decade, this species has invaded natural communities of meadows and forest edges. The high rate of expansion of the secondary distribution range, the reduction of natural diversity in regions, as well as direct damage caused by this species when laying lawns, allow as to include *V. filiformis* in the list of potentially invasive alien species and to take measures to control its dispersal [188].

The *Oenothera* L. genus is the best model taxon to study microevolutionary changes accompanying intercontinental invasions from America to Europe. *Oenothera* have a special breeding system (constant translocation heterozygosity) [189], which promotes hybridization (including introgressive hybridization) between any species, leading to the formation of hybrids with a constant combination of morphological characteristics. An analysis of hybrid species distribution in Europe proves the existence of species with different degrees of invasiveness [190]. A positive correlation has been found between the degree of invasiveness displayed by parental species and the invasiveness of their hybrids. As a rule, noxious hybrids descended from noxious parents. The most noxious hybrids were formed after crossing North American and European species. The invasiveness of morphologically similar hybrids correlates both with their genetic traits and the abundance of parental species. The species invasiveness also depends on the plant cytotype [190]: species with chromosome rings display a higher invasiveness compared with those with bivalent chromosomes.

### 3.8. Identifying the Biological Characteristics of Invasive Species

This approach is important when dealing with noxious plants. Among the research papers we have studied, the most interesting ones are those in which invasive and non-invasive alien species or native ones are compared in terms of detecting features allowing scholars to predict whether one or another species will become invasive. Such studies have been performed for species from the genera *Conyza* Less., *Galega* L., *Robinia* L., *Lupinus* L., *Caragana* Lam., and *Impatiens* L.

*Conyza bonariensis* (L.) Cronq. displays competitive advantages over *C. canadensis*: this species forms twice as many diaspores, being tolerant to dry poor soils and having dense pubescence. All of the above allows it to grow in arid habitats with intensive illumination. On the other hand, *C. canadensis*, being of a more northern origin, displays higher tolerance to low temperatures and is adapted to long daylight hours, which enables it to expand to the north. However, we cannot exclude the possibility of further northward movement of *C. bonariensis*, bearing in mind its further adaptation and climate changes [191]. During our study, several individual plants could not be attributed to any species based on analysis of morphological features (inflorescences shape and diameter and the shoots and leaves pubescence degree) since they had intermediate parameters. In order to analyze the hybridogenic activity of *Conyza* in the secondary distribution range, we have applied molecular genetic analysis to ISSR fragments and the ITS site of nuclear DNA. All *Conyza* invasive species have a very low hybridogenic activity, and plants with intermediate traits are not always hybrids.

The variability of 18 morphometric characteristics was revealed in the study of *Conyza canadensis* in indigenous and anthropogenic ecotopes in the southwestern part of the Middle Russian Upland as early as in 2011 [192]. Populations formed in anthropogenic ecotopes were most original. The average correlation relationship in native, quasi-natural, and anthropogenic ecotopes increased along with a rise in anthropogenic influence. Visualization of the correlation using factor analysis allows us to speak about changes in morphological characteristics of *Conyza canadensis* depending on the degree of anthropogenic influence [193].

### 3.9. Comparative Analysis of the Invasive Status of Closely Related Alien Species

A methodology for the analysis was developed in 2014, based on the example of the Leguminosae family [194]. Legumes (Fabaceae/Leguminosae) are among the leaders in bringing about harmful consequences of plant invasions. In Central Russia, legumes occupy the fifth place in the number of alien species (79 in total, 43 naturalizing ones). The large-scale dissemination of the legume species may be explained by their mass usage in agriculture as forage grass as well as soil fertility boosters. Benevolent intentions soon demonstrate the opposite side of “environmental improvement”: invading a habitat, lacking nitrogenous compounds; legume species fertilize soil with nitrogen, making it suitable for other alien weeds. Since all the changes occur at the ecosystem level, even a complete elimination of the invasive legume species would not return the community to its initial (“pre-invasion”) status.

Within the last 20 years, the alien fraction of flora in Central Russia has gained 80 new species of Fabaceae. About 20 of them have been brought accidentally, 30 are increasing their natural distribution range to the north, and the other 30 are represented with the species which have escaped from cultivation. Fast-spreading invasive legume species belong to the third group—*Lupinus polyphyllus* Lindl., *Galega orientalis* Lam. and *Robinia pseudoacacia* L., as well as the actively naturalizing and potentially invasive *Amorpha fruticosa* L. and *Caragana arborescens* L. These five species are still widely cultivated, so we expect a further increase in their secondary distribution range and invasive status.

*Lupinus polyphyllus* has a competitive advantage over closely related *L. angustifolius* L. in more numerous beans and the number of seeds per plant, a larger leaf surface (both of individual leaflet and general surface of compound leaf), more numerous flowers per inflorescence, and androecium capability, allowing two anthesis periods for individual flowers [194]. *L. polyphyllus* has a vegetative propagation capacity and *L. angustifolius* is an annual. Thus, in another few characteristics *L. polyphyllus* is inferior to *L. angustifolius,* having smaller beans and seeds and a smaller number of lateral shoots and leaves per shoot, which results in less assimilating surface. High pollen fertility and germinating capacity of scarified seeds is typical of both species.

*Galega orientalis* has a competitive advantage over closely related *G. officinalis* L. in more numerous flowers and seeds per inflorescence, higher pollen fertility, vegetative propagation capabilities, and higher population density [195]. The main characteristic feature of *Galega orientalis* is its cultigeneous distribution range, far exceeding the one for *G. officinalis.* On the other hand, *G. orientalis* is inferior to *G. officinalis* in the number of lateral shoots per plant and germinating capacity of scarified seeds. There are no significant differences between these species in the number of leaves per generative shoot, bean length, the number of seeds per bean, the structure and development of generative sphere (excluding its smaller size in *G. officinalis*).

Invasive *Robinia pseudoacacia* has a competitive advantage over cultivated *R.× ambigua* Poir. in the number of seeds per bean, the number of flowers/fruits per inflorescence, slightly larger pollen grains, and significantly (2.5 times) higher pollen fertility [194]. On the other hand, *R. × ambigua* flowers and fruits a few times within a season, whereas reflorescence does not occur in *R. pseudoacacia* every autumn (and fruit inception does not happen at all).

*Amorpha fruticosa* has a competitive advantage over cultivated *A. paniculata* Torr. and *A. californica* Nutt. in earlier phenological phases, higher seed production, seed germination capacity, earlier seed sprouting, seedlings growth dynamics within the first year, and significantly larger cultigeneous distribution range.

*Caragana arborescens* has a competitive advantage over closely related *C. arborescens* f. *lorbergii* Koehne and *C. laeta* Kom. in larger pollen-grains, more numerous flowers per inflorescence, much higher seed germination capacity, and long-standing cultivating tradition, resulting in significantly larger secondary distribution range. Thus, Central Asian *C. laeta* has larger flowers [194].

While comparing the plants of *G. orientalis* from natural and secondary distribution ranges, we obtained data supporting the Evolution of Increased Competitive Ability hypothesis. The invasive phenotype appeared more “powerful” than the natural one: the biomass of above-ground organs, inflorescence length, the number of flowers/fruits per plant, and seed production exceed significantly within the secondary distribution range.

Thus, in all the compared pairs of species, the invasive ones have a competitive advantage over closely-related non-invasive ones in more numerous flowers/fruits in raceme and denser populations.

To test the hypothesis of a competitive superiority displayed by invasive species, we compared the invasive *Impatiens parviflora,* invasive *I. glandulifera* Royle, naturalized *I. nevskii* Pobed., and native *I. noli-tangere* L. in terms of morphometric traits of their flowers at different development stages. A number of features in which alien species display a competitive superiority over closely related *I. noli-tangere* have been revealed. The morphological variability has been defined by morphometric investigations of the 27 characteristics. There is a tendency for alien *Impatiens* species toward an earlier development of androecium and gynaeceum: caliptra is formed at the stage of uncolored bud, the pistil is differentiated in ovary, short style and stigma—at the stage of colored bud. We have not identified any other morphometric flower traits which would offer more competitiveness of invasive *I. glandulifera* and *I. parviflora* compared with the native *I. noli-tangere* and naturalized *I. nevskii* [196].

Invasive species also far exceed non-naturalizing ones in the area of cultigeneous distribution range. The invasive activity of one and the same species in different regions within secondary distribution range varies greatly, which is determined by both natural and anthropogenic factors: species invade more actively in natural plant communities within the regions where they have been widely cultivated for a long time [197].

The study of herbarium material (FR, B, PRA, IB PAN KTU, LE, MW, MHA, MOSP, BSU, RV, KW, DNZ) and field studies carried out in Germany, France, Holland, Poland, Czech Republic, Slovakia, Ukraine, and Russia reveal the distribution pattern of *Oenothera* spp. We have found that species with large, medium, and small flowers occupy different habitats [167]. As it turns out, species with medium-sized flowers are most adapted to the climatic conditions of Europe, they have a wide ecological amplitude and are therefore most widely distributed [167]. The complex of *Oenothera* spp. in Eastern Europe is divided into groups according to the degree of invasiveness.

### 3.10. Studying Micromorphological Characteristics in Invasive Species and Assessing Their Significance for Taxonomy

We have studied achenial trichomes of 23 taxa belonging in three genera of Asteraceae, using digital and scanning electron microscopy (SEM): nine intraspecific taxa of *Solidago*, seven of *Conyza*, and seven of *Bidens*. The research shows that achenes of all the *Conyza* and *Solidago* taxa examined are covered with duplex trichomes. This feature is variable within *Bidens*: indigenous European species *B. tripartita* L. and *B. cernua* L. are characterized by simple monostichous multicellular trichomes; and the invasive *B. frondosa*—by duplex trichomes, the invasive *B. connata* Muhl. ex Willd.—by trichomes of both types. Additional characteristics of taxonomic value have been described for several taxa: trichome length, pubescence, surface sculpture, etc. Identification keys based on trichome characters have been created for species of *Bidens* and *Solidago* [198].

We have also compared the morphometric features inherent in the stomatal apparatus of cultivated *Symphyotrichum* Nees species in order to assess the adaptive capacity of these alien taxa. Eleven species of *Symphyotrichum* have been studied, including two hybrid ones [199]. According to the index of relative transpiration area, the species are divided into three groups: those with a high (12–14%) relative transpiration area (*S. novae-angliae* (L.) G.L. Nesom, *S. novi-belgii* (L.) G.L. Nesom, and *S. × salignum* (Willd. (pro sp.)) G.L. Nesom); medium (3–7%) area (*S. lateriflorum* (L.) Á. Löve & D. Löve, *S. ciliolatum* (Lindl.) Á. Löve & D. Löve, *S. leave* (L.) Á. Löve & D. Löve, *S. × versicolor* (Willd. (pro sp.)) G.L. Nesom, and *S. puniceum* (L.) Á. Löve & D. Löve); and low (0.2–2.0) relative transpiration area (*S. chilense* (Nees) G.L. Nesom, *S. cordifolium* (L.) G.L. Nesom, and *S. tradescantii* (L.) G.L. Nesom). Similar data were obtained earlier for the genus *Solidago* and *Impatiens*: a positive correlation between the relative transpiration area and the alien species invasiveness was revealed. Therefore, we suggest the following hypothesis: a higher index of the relative transpiration area indicates a greater alien species adaptability and can be used, along with other features, to predict further expansion of their secondary distribution range and increase in the chances of their transformation into invasive species.

### 3.11. Studying the Phytochemical Characteristics of alien Species

Useful wild plants usually decrease the content of biologically active substances in the culture. However, no research has been done on the reverse process. Therefore, we do not know whether the level of biologically active compounds increases in plants “escaping” from cultivation and invading natural communities (invasive species). We have studied *Aronia melanocarpa* (Michx.) Elliot, *A. arbutifolia* (L.) Pers., and *A. × prunifolia* (Marshall) Rehder in the arboretum of the Main Botanical Garden (Moscow, Russia), which were brought from the USA in the 1980s. Two samples of cultivated *A. mitschurinii* A.K. Skvortsov et Maitulina and one sample of invasive *A. mitschurinii* from the Moscow region were also included in the analysis. At this stage of the study, we aimed at determining the degree of heritability of macro- and micromorphological characteristics of North American plants introduced to Europe and comparing them with analogous parameters of cultivated *A. mitschurinii,* especially with naturalized plants. Our research purpose also included identification of samples most promising for further broad cultivation due to their antioxidant activity and the content of microelements in leaves. We examined the trichome density and stoma parameters of leaves using a Keyence VHX-1000 digital microscope and a LEO 1430 VP scanning electron microscope. The 2,2-difenyl-1-picrylhydrazyl (DPPH) free radical scavenging test was used in order to determine the antioxidant activity of the fruits. The content of microelements in the plant material was determined by using ICP-MS (Agilent 7700ce, Santa Clara, CA, USA). Thus, the diagnostic features of the introduced North American *Aronia* proved to be inherited in cultivation. The fruit mass increases in the following order: *A. arbutifolia* → naturalized *A. mitschurinii* →*A. × prunifolia* → *A. melanocarpa* → cultivated *A. mitschurinii.* An original table has been compiled to compare the studied taxa in 21 bio-morphological features. The total antioxidant activity in dry fruits has been determined for methanol, ethanol, and for water extracts. Antioxidant activity is higher in alcohol extracts of naturalizing plants than in those of cultivated ones. On the contrary, the antioxidant activity of water extracts is lower in the case of naturalizing plants. There are 17 microelements (ppm) in the leaves of *Aronia* taxa. *A. mitschurinii* have the highest content of 10 microelements: Fe, Mn, Sr, Zn, Se, Cu, Mo, Cr, As, and Sb; *A. × prunifolia* have the highest content of 6 ones: Ni, Co, V, Cd, Pb, and Sn; and *A. arbutifolia* have the highest content of B. Our observations suggest that naturalizing plants of *Aronia* represent a potential source of useful bioactive compounds. Apparently, plants accumulate fewer biologically active substances in a comfortable environment than during forced adaptation to unfavorable ecologic conditions [200].

In our 2017 research, we studied the chemical composition of oil in seeds of *Echinocystis lobata* to reveal that ripe seeds contained more than 20% of fatty oil. Oil composition in both populations under study varied insignificantly in terms of quantity [201]. Fatty acids were mostly represented by the following six ones: palmitic, stearic, oleic, linoleic, linolenic, and hexanedioic acid. Arachidic and behenic acid were detected in negligible amounts (less than 1%). The oil had a low content of oleic acid (ca. 12%), though it was rich in polyunsaturated fatty acids (linoleic and linolenic acid). The major component of the oil in *E. lobata* seed was linoleic acid (up to 61%). In our experiment, linolenic acid content was found to be up to 2%, while the percentage of saturated fatty acids (palmitic and stearic, ca. 18%) appeared to be relatively low, compared with respective percentages in other fatty oils of vegetable origin, such as cotton, sea buckthorn, pumpkin, or soy oil.

For the purposes of this paper, we have assessed the antioxidant activity of *Solidago* L. complex applying the DPPH method to the dried plant raw material (leaves and inflorescences) [202]. We compared 13 samples grown from seeds at the experimental plots in the vicinity of Moscow belonging to seven species: *Solidago virgaurea* L., *S. altissima* L. (=*S. canadensis* s.l.), *S. canadensis* L. s.str, *S. gigantea* Aiton, *S. × niederederi* Khek. (=*S. virgaurea × S. canadensis*), *S. × snarskisii* Gudžinskas & Žalneravičius (=*S. virgaurea × S. gigantea*), and *S. graminifolia* (L.) Nutt. (=*Euthamia graminifolia* (L.) Nutt.). The seeds were collected in Hungary, Belarus, Russia, and the Czech Republic. The antioxidant activity of alcohol extracts was very high in all specimens. The total antioxidant activity in the leaves amounted to 75.28–92.77% (methanol extracts), 44.34–91.52% (ethanol extracts), and 21.55–68.51% (water extracts). The total antioxidant activity in the inflorescences was 90.77–94.07% (methanol extracts), 90.20–95.66% (ethanol extracts), and 37.69–64.47% (water extracts). In all experiments, antioxidant activity was 2.5–3.0 times higher in alcohol extracts than in aqueous ones. The lowest antioxidant activity both in alcohol and aqueous extract was shown by *S. snarskisii* (leaves). This taxon, in general, displays the greatest variability of morphological and biochemical characteristics, which apparently reflects its hybridity. Alcohol extracts from the leaves of both *S. graminifolia* samples also showed a reliably lower antioxidant activity (84 vs. 91% in methanol and 85 vs. 90 in ethanol), which to some extent indirectly confirmed the correctness of referring it to the *Euthamia* genus. Consequently, in terms of antioxidant activity, alien *Solidago* species and their hybridogenic species *S.* × *niederederi* are similar to the native *S. virgaurea* included in the official pharmacopeia.

### 3.12. The Influence of Hybridization Processes on Alien Plant Species Invasion Rate

We have taken an inventory of invasive species, including those of hybridogenic origin, in various regions and habitats. The hypothesis of a hybridogenic origin of *Bidens* × *decipiens* Warnst. has been tested using ISSR analysis to reveal that *B. × decipiens* can be considered as a complex of hybrids and backcrosses of the native *B. cernua* and the alien *B. frondosa*. Analysis of the ITS nuclear and trnL-trnF chloroplast sections has confirmed the hybridogenic origin of this taxon and thus allows us to establish that the maternal species was *B. cernua* and the paternal one was probably *B. frondosa*. Previously, floristic summaries referred this taxon to the North American alien species *B. connata*. The name “*B. connata*” is indicated as ineligible for a taxon that does not grow in North America. Contrary to the hypothesis explaining the success of plant invasion in a new homeland by strengthening of hybridization processes in the secondary range [2], *Bidens × decipiens* has proved to display a very low hybridization activity in Europe and to be less competitive than the parent species, the North American *B. frondosa*.

We have found that the invasive *Conyza bonariensis* does not display any hybridogenic activity in Southern Europe, or its level is rather low, whereas *C. sumatrensis* and *C. canadensis* can sometimes hybridize with each other. The number of hybrids and backcrosses has been insignificant so far, but without doubt, further study of hybrid activity of *Conyza* is necessary to predict the expansion of their secondary distribution range and possible threat to the biodiversity of the Mediterranean region. This is all the more relevant since in 2018 we found a stable invasive population of *C. sumatrensis* (Retz.) E.H. Walker. in the Crimea (prior to that, Sochi was the only territory where this species grew in Russia).

Another object of our study included diagnostic morphological characteristics of three *Solidago* taxa growing together in the vicinity of Pskov: a native *S. virgaurea*, an invasive species of North American origin *S. canadensis* and their hybrid *S. × niederederi*. *S. × niederederi* has an intermediate position between *S. virgaurea* and *S. canadensis* in terms of head diameter, panicle compactness degree, and area of rosette leaves, and is similar to *S. canadensis* in degree of pubescence. The hybrid origin of *S. × niederederi* has been proved by molecular analysis of nuclear DNA nucleotide sequences (ITS site). It is not yet possible to unambiguously answer the question of which parent species is maternal and which is paternal. The analysis of high-variable non-coding areas of chloroplast DNA (rpl32-trnL) suggests that hybridization proceeds in both directions.

We have predicted the rate of further invasion of the *Solidago* L. hybridogenic taxa with the verification of their hybridogenic character using experimental and molecular-genetic methods. Not all taxa with intermediate morphological characteristics are hybrids. Hence, neither experimental nor molecular-genetic methods have confirmed the hybrid origin of “*Solidago canadensis × S. gigantea*”. The *Solidago × niederederi* and *S. × snarskisii* species occurring in Europe have been found in the secondary distribution range of the parental species only sporadically, and they are less competitive than their ancestors. The tendency toward naturalization is displayed by *S. × niederederi* only.

Thus, the example of three genera of the Asteraceae family shows that the hypothesis of higher competitiveness and invasiveness of hybrids compared with parental species is not comprehensive, and the examples supporting it are an exception rather than the rule [203].

### 3.13. Removing the Most Invasive Species for Experimental Purposes

We have conducted experiments to reduce the abundance of invasive species. The species under examination were represented with *Impatiens glandulifera* Royle (annual), *Solidago gigantea* Ait. (long rhizome), and *Adenocaulon adhaerescens* Maxim. (short rhizome), growing in masses on the territory of the Main Botanical Garden (Moscow). During the study, experimental plots of 2 × 1 m were established. A small plot size was adequate for sampling relatively species-poor herbaceous plant communities. The plots had a high covering of the species examined (80–100%). The vegetation composition was assessed using frequency measures (shoots/m^2^) of the investigated species and accompanying ones. Each experimental plot was further divided into two parts: one of them (1 × 1 m) was assigned to a removal treatment, while the remaining part was left unmanipulated (the control plot). During the following 2–3 years, the changes occurring on experimental plots were recorded and invasive species were removed repeatedly.

For *Impatiens glandulifera,* experimental plots were established: (1) along the Likhoborka river, (2) on a flood plain under willow curtains, and (3) in a small fen with *Phragmites communis*. Our attempt to reduce the *I. glandulifera* abundance failed. On the control plots, density of stands in 2006–2008 varied insignificantly (200–350 plants/m^2^), depending on ecotope characters, while on the plots where removal was made, the density of stands increased year after year to achieve approximately 700 plants/m^2^ in 2008. Native species were not found on the plots, however seedlings of another noxious species—*Heracleum sosnowskyi* Manden.—emerged.

Better results were received for *S. gigantea* (supervision 2007–2009). Experimental plots were established: (1) on the wasteland, (2) on a margin of an oak grove, and (3) on a pond coast. As a result of the treatment, photon flux density at the ground level was 6–10 times higher on the removal plots than on control ones. On the first plot, the shoot number of *S. gigantea* decreased 2.5 times (101 vs. 260) and the number of native species increased (7 vs. 4) in two years. On the second plot, the shoot number of *S. gigantea* decreased 1.2 times (144 vs. 179) and the number of native species also increased (6 vs. 2). On the third plot, the shoot number of *S. gigantea* increased 1.2 times (153 vs. 131). Nevertheless, the number of native species increased (11 vs. 6), and two other invasive species (*Geum macrophyllum* and *Impatiens parviflora*) emerged.

The greatest success was achieved in eradication of *Adenocaulon adhaerescens*. This species “escaped” from cultivation about 15 years ago. It has widely extended along garden roads and footpaths. The density of its stands varies from 83 to 211 plants/m^2^ (133.4 ± 35.8 on average). No native species can grow on such sites. In 2009, as many as 24,692 individual plants (about ¾ growing in a garden) were destroyed. The following year, the number of plants on all experimental plots decreased ten times (473 vs. 4247) and some native species appeared, such as *Galeobdolon luteum* Huds., *Aegopodium podagrarium* L., *Impatiens noli-tangere* L., etc. [204].

### 3.14. Invasive Species as Resource Plants

Many invasive species can become new resource plants. The long-established cultivation of species which have now become invasive indicates that they have valuable properties. We have collected data on the medicinal, melliferous, food, silage, and fodder benefits offered by invasive species, summarizing the global experience of using invasive alien plants [205,206]. Our findings suggest collecting invasive species in wild-growing invasive populations (not cultivated ones!) and using them as food, medicines, dyestuffs, and so on. Thus, when harvesting invasive species in natural ecosystems, we use significant reserves of economically valuable raw materials and reduce the negative impact of alien plants on the region’s biodiversity.

We should also pay attention to invasive species with ornamental properties. In addition, a reasonable warning must be made about restricting their cultivation and strictly observing the techniques for utilizing plant residues. A high degree of invasiveness in some alien species and the danger of their uncontrolled spread also deserves consideration. Research conducted by institutes of the Russian Academy of Sciences provides substantial data on the chemical composition of medicinal species, especially the biologically active flavonoid complex and silicon compounds.

Without doubt, many invasive plants have properties which are valuable for humans. However, it is equally important to assess the risk posed by the presence of such species in regional ecosystems and to take a clear stand in respect to them. More and more experts stress the need to restrict their cultivation. Quite a number of EU states legally ban any use of certain invasive species and require their eradication. Similar policies are now prepared to be approved in various regions of Russia. Among the examples of relevant measures taken in Russia from 2011–2017, there were at least 477 contracts for eradication of *Heracleum sosnowskyi* thickets to a total amount of 314 million rubles. Unwanted vegetation in the area of 169 thousand hectares was mapped, and an area of 18 thousand hectares was cleared from plant thickets. Most large-scale projects aimed at eradication of *H. sosnowskyi* were conducted in Leningrad, Moscow, and Vologda regions, where funds necessary to fight plant invasion were included in regional budgets [207].

### 3.15. Glossary Compilation

In 2018, research on “The main terms and concepts used in the study of alien and synanthropic flora” was published [208]. As of today, notes have been added to a number of terms containing the etymology of names and interpretation used in international or domestic literature, corresponding to other classifications of alien plant species.

### 3.16. Studying the Consortial Relationships between Invasive Species and Pathogens, Phytophages, and Pollinating Insects

The evolution of the consortial relationships system and the role it plays in further expansion of introduced species deserve scientific attention. As part of this task, in 1990–2010 we carried out a systematic monitoring of phytophages and pathogenic organisms of *Ribes aureum* Pursh. The findings suggest that during naturalization, pathogens adapt to alien plants. The duration of this process is determined by environmental conditions, the effect of anthropogenic factors, and the age of the plantings. Most phytophages and phytopathogens are also found on other species of the genus *Ribes* L. Thus, the hypothesis concerning the effect of phytophages and phytopathogens on the success of alien species invasions in the secondary range may only be considered at the initial stage of naturalization. In future, phytophages and pathogens of closely related species are actively included in the “alien species—pathogen” system [179].

### 3.17. Creating a System to Track Invasive Species Distribution Online

This task is a remote one, only the first steps are being taken towards its implementation. For instance, the website at https://www.inaturalist.org/projects/flora-of-russia; (accessed on 24 June 2021) allows the public to post observations on the occurrence of invasive species in regions. iNaturalist is a joint initiative of the California Academy of Sciences and the National Geographic Society supported by dedicated personnel and developed by the scientific and civil communities. Photos of plants with coordinates of respective localities are regularly published on the platform which supports mapping distribution ranges of native and invasive species. We successfully use iNaturalist for studying the secondary distribution ranges of alien species and their distribution dynamics, for example, *Adenocaulon adhaerescens.* This species was introduced in living plant collections of the Main Botanical Garden Russian Academy of Sciences (MBG) from seeds collected in 1953 within Vladivostok city limits. After 30 years, we recorded several plants which had developed from seeds spontaneously, beyond the exposition of the Russian Far East. In 1980s, *A. adhaerescens* plants were found in masses along the Likhoborka river valley. This species was first recorded beyond MBG territory in 1997.

By the end of the 20th century, *A. adhaerescens* had become an obtrusive weed within the MBG territory. In 2005, *A. adhaerescens* was recorded in the Ostankino Park and VDNKh (Exhibition of Achievements of the National Economy), that is, in areas adjacent to MBG; in 2007 it was recorded in the West of Moscow, very far from MBG territory [209].

Data found on iNaturalist have made it possible to reveal new localities/populations of *A. adhaerescens* in Moscow and Moscow region. Eight habitats have recently been found within Moscow city limits and in Moscow region. In populations of *Adenocaulon adhaerescens* along trails in parks and recreational forests, its projective cover may reach 100%. The biggest plants produce more than 5000 achenes per season equipped with viscid glandules. These glandules ensure effective seed distribution over big distances (both via anthropo- and zoochory). A spontaneous introduction of this species in East European countries and its further invasion in natural plant communities is possible. Therefore, we need to monitor its initial populations and take measures to control its distribution [210].

## 4. Conclusions

The rapid rate of new invasive species emergence and their introduction into natural biocenoses have triggered the development of invasive biology in Russia. In almost 40 years, researchers have gone from a few finds of alien species to compiling a complete list of invasive plants, clarifying the most important invasion pathways and determining the rate of microevolutionary changes of species in the secondary distribution range. Now it is necessary to focus on controlling the dispersal of invasive species in the country.

The aspects of alien species analysis outlined in the paper are of great importance for the development of invasive biology. Many studies have been integrated, and this approach will help us understand why only a few of the alien species are transformed into invasive ones. Since the most active alien species disperse without regard to national borders, we consider it important to coordinate and to integrate specialists from different countries for joint studies of invasive species in order to promote the development of the research trends discussed in our review. Furthermore, it will enable scholars to obtain significant joint findings concerning other issues requiring scientific attention. These may include the impact of global climate change on the flora dynamics and its alien fraction, the influence of transportation network development on the dissemination of alien and indigenous species, and so on. The case of the xerophytized flood plain of the Desna River (in Bryansk region) is a good example of the climate change effect on vegetation. In particular, warmer temperatures recorded in the area in 2018–2020 enhanced the phytocenotic activity of *Acer negundo, Bidens frondosa, Erigeron annuus* (L.) Pers., *Conyza canadensis, Epilobium adenocaulon* Hausskn., and *Epilobium pseudorubescens* A.K. Skvortsov.

There is also a growing interest toward the study of rare indigenous plants occurring in the vicinity of railways [211,212]. An analysis of the interaction between various flora fractions may be key to understanding the mechanisms of ecosystem transformations.

## Figures and Tables

**Table 1:** The study of alien and invasive flora fractions in various administrative subjects of Russia.

Administrative Subjects of Russia	Area, ths. km^2^	Area, % of Country	Papers on Alien Plant Species	Papers on Invasive Plant Species
Central Federal District				
Belgorod Region	27,134	0.16	[33,49,50]	[24,51,52]
Bryansk Region	34,857	0.20	Data are fragmentary	[52,53]
Vladimir Region	29,084	0.17	[44]	[52]
Voronezh Region	52,216	0.30	[39]	[52,54,55,56]
Ivanovo Region	21,437	0.13	[43,44]	[52,57]
Kaluga Region	29,777	0.17	[45]	[52,58]
Kostroma Region	60,211	0.35	[59]	[52]
Kursk Region	29,997	0.18	[60]	[52]
Lipetsk Region	24,047	0.14	[61]	[52]
Moscow Region	44,329	0.26	[13,14,15,16,22,48]	[17,48]
Orel Region	24,652	0.14	Data are fragmentary	[52]
Ryazan Region	39,605	0.23	[62]	[52]
Smolensk Region	49,790	0.29	Data are fragmentary	[52]
Tambov Region	34,462	0.20	Data are fragmentary	[52]
Tver Region	84,201	0.49	[35,40,44,46,63]	[52,63,64]
Tula Region	25,679	0.15	[37,65]	[52]
Yaroslavl Region	36,177	0.21	[44,66]	[52,67,68]
The City of Moscow	2,561	0.01	[19,48]	[48]
Total	650,205	3.78	All the regions have been explored sufficiently	
The North-West Federal District				
Republic of Karelia	180,520	1.05	[5]	[69]
Republic of Komi	416,774	2.3	[70]	No data available
Arkhangelsk Region	589,913	3.44	No data available	No data available
Vologda Region	144,527	0.84	No data available	No data available
Kaliningrad Region	15,125	0.09	[8]	[71]
Leningrad Region	83,908	0.49	[5,6,7]	[69]
Murmansk Region	144,902	0.85	No data available	No data available
Novgorod Region	54,501	0.32	Data are fragmentary	[69]
Pskov Region	55,399	0.32	Data are fragmentary	[69]
The City of Saint Petersburg	1403	0.01	[72]	No data available
Total	1,686,972	9.71	7.44% of the territory remain underexplored	
The South Federal District				
Republic of Adygeya	7792	0.05	[73]	Data are fragmentary
Republic of Daghestan	50,270	0.29	No data available	No data available
Republic of Ingushetia	3123	0.02	No data available	No data available
Kabardino-Balkarian Republic	12,470	0.07	No data available	[74]
Republic of Kalmykia	74,731	0.44	No data available	No data available
Karachayevo-Circassian Republic	14,277	0.08	No data available	No data available
Republic of North Ossetia-Alania	7987	0.05	No data available	[75]
Chechen Republic	16,171	0.09	No data available	No data available
Krasnodar Territory	75,485	0.44	No data available	[76,77]
Stavropol Territory	66,160	0.39	No data available	No data available
Astrakhan Region	49,024	0.29	[78]	Data are fragmentary
Volgograd Region	112,877	0.66	[79]	[80]
Rostov Region	100,967	0.59	No data available	No data available
Total	447,821	3.46	1.95% of the territory remain underexplored	
The Privolzhsky (Volga) Federal District				
Republic of Bashkortastan	142,947	0.83	No data available	[25,26]
Republic of Mariy El	23,375	0.14	[10]	
Republic of Mordovia	26,128	0.15	[28,38]	[81]
Republic of Tatarstan	67,847	0.40	No data available	No data available
Udmurt Republic	42,061	0.25	[36]	[82]
Chuvash Republic	18,343	0.11	No data available	No data available
Kirov Region	120,374	0.70	[27,83]	No data available
Nizhny Novgorod Region	76,624	0.45	[83]	[84,85]
Orenburg Region	123,702	0.72	No data available	No data available
Penza Region	43,352	0,25	Data are fragmentary	[86]
Perm Region	160,236	0.94	No data available	No data available
Samara Region	53,565	0.31	Data are fragmentary	[30,87]
Saratov Region	101,240	0.59	[88]	No data available
Ulyanovsk Region	37,181	0.22	[47]	[30,87]
Total	1,036,975	6.06	3.60% of the territory remain underexplored	
The Ural Federal District				
Kurgan Region	71,488	0.42	No data available	No data available
Sverdlovsk Region	194,307	1.13	[89]	[90]
Tyumen Region (without autonomousareas)	160,122	0.94	[91]	
Khanty-Mansi autonomous area—Yugra	534,801	3.12	No data available	No data available
Yamalo-Nenets autonomous area	769,250	4.49	No data available	No data available
Chelyabinsk Region	88,529	0.52	No data available	No data available
Total	1,818,497	10.62	9.49% of the territory remain underexplored	
The Siberian Federal District				
Republic of Altai	92,903	0.54	[92]	[93]
Republic of Tyva	168,604	0.98	[94]	[93]
Republic of Khakassia	61,569	0.36	[95]	[93]
Altai Territory	167,996	0.98	[91,96,97]	[93]
Krasnoyarsk Territory	2,366,797	13.82	[98]	[93]
Irkutsk Region	774,846	4.52	[99]	[93]
Kemerovo Region	95,725	0.56	[100]	[93,101]
Novosibirsk Region	177,756	1.04	[42,91]	[93]
Omsk Region	141,140	0.82	[91]	[93]
Tomsk Region	314,391	1.84	[102,103]	[93]
Total	4,361,727	25.46	All the regions have been explored sufficiently	
The Far Eastern Federal District				
Republic of Sakha (Yakutia)	3,083,523	18.01	[104,105,106,107,108,109]	[106]
Primorsky Territory	164,673	0.96	[110,111,112,113,114,115,116,117,118,119,120,121,122,123,124,125,126]	[105,127,128]
Khabarovsk Territory	787,633	4.60	[129,130,131,132,133]	[105,130,131,132]
Amur Region	361,908	2.11	[134,135,136,137]	[105,138]
Kamchatka Territory	464,275	2.71	[139,140,141]	[105,142,143]
Magadan Region	462,464	2.70	[144,145,146,147,148]	[105]
Sakhalin Region	87,101	0.51	[149,150]	[105,151]
Jewish Autonomous Region	36,271	0.21	[152,153]	[105]
Chukotka autonomous area	721,481	4.21	[154]	[105]
Republic of Buryatia	351,334	2.05	[155,156,157]	[93,105]
Trans-Baikal Territory	431,892	2.52	[158]	[93,105]
Total	6,952,555	40.59	All the regions have been explored sufficiently	

Note: Data from reviews and major special papers have been used to compile the Table.

## Data Availability

The data presented in this study are available in article.
